# Estrogens in polycystic liver disease: A target for future therapies?

**DOI:** 10.1111/liv.14986

**Published:** 2021-07-10

**Authors:** Sophie E. Aapkes, Lucas H. P. Bernts, Thijs R. M. Barten, Marjan van den Berg, Ron T. Gansevoort, Joost P. H. Drenth

**Affiliations:** ^1^ Department of Nephrology University Medical Center Groningen University Hospital Groningen Groningen the Netherlands; ^2^ Department of Gastroenterology and Hepatology Radboud University Medical Center Nijmegen the Netherlands; ^3^ Department of Gynaecology University Medical Center Groningen University Hospital Groningen Groningen the Netherlands

**Keywords:** ADPKD, estrogen, GnRH analogues, polycystic liver disease, progesterone, tamoxifen

## Abstract

**Background and Aims:**

Patients suffering from polycystic liver disease (PLD) can develop large liver volumes, leading to physical and psychological complaints, reducing quality of life. There is an unmet need for new therapies in these patients. Estrogen seems to be a promising target for new therapies. In this review, we summarize the available experimental and epidemiological evidence to unravel the role of estrogens and other female hormones in PLD, to answer clinical questions and identify new targets for therapy.

**Methods:**

We identified all experimental and epidemiologial studies concerning estrogens or other female hormones and PLD, to answer pre‐defined clinial questions.

**Results:**

Female sex is the most important risk factor for the presence and severity of disease; estrogen supplementation enhances liver growth and after menopause, liver growth decreases. Experimental studies show the presence of the estrogen receptors alfa and beta on cystic cholangiocytes, and increased in vitro growth after administration of estrogen.

**Conclusions:**

Based on the available evidence, female PLD patients should be discouraged from taking estrogen‐containing contraceptives or hormone replacement therapy. Since liver growth rates decline after menopause, treatment decisions should be based on measured liver growth in postmenopausal women. Finally, blockage of estrogen receptors or estrogen production is a promising target for new therapies.

**Abbreviations**
ADPKD: autosomal dominant polycystic kidney disease
ADPLD: autosomal dominant polycystic liver disease
BDL‐rats: bile duct‐ligated rats
CT: computed tomography
EGF: epidermal growth factor
EGFR: epidermal growth factor receptor
ER: estrogen receptor
FSH: follicle stimulating hormone
FSHR: follicle stimulating hormone receptor
GNRH: gonadotropin releasing hormone
GPER1: G‐coupled protein estrogen receptor 1
hTLV: height‐adjusted total liver volume
IGF: insulin like growth factor
LH: luteinizing hormone
RI: magnetic resonance imaging
TLV: total liver volume

**Key points**

- Female gender is the most important risk factor for polycystic liver disease.
- Estrogen receptors are present on human hepatic cyst cholangiocytes.
- In bile duct‐ligated rats and in in vitro human hepatic cell lines, cholangiocyte growth increases after administration of estrogen and decreases after administration of estrogen blockers (tamoxifen or fulvestrant).
- Estrogen seems to be a promising target for new therapies.
- Estrogen inhibition can be approached in two ways: on a receptor level using selective estrogen receptor degraders or moderators, or on a systemic‐level blocking estrogen production.

## 1 INTRODUCTION

Polycystic liver disease (PLD) is characterized by enlargement of the liver due to the growth of numerous liver cysts.^1^ It can be caused by either Autosomal Dominant Polycystic Liver Disease (ADPLD), a cystic disease affecting the liver only, or Autosomal Dominant Polycystic Kidney Disease (ADPKD), which also causes cyst formation in the kidneys and renal function decline. The prevalence of ADPKD is 1:2500.^1^, ^2^ Most ADPKD patients have at least some liver cysts but about 10% of ADPKD patients suffer from severe PLD (a height‐adjusted total liver volume (hTLV) >3200 ml).^3^ ADPLD is more rare with an incidence of 1:158 000.^4^ Together, we estimate that about 1:20 000 patients suffer from severe PLD, caused by either ADPLD or ADPKD.

Although liver function remains well‐preserved in most patients, the unrestricted growth of liver cysts can lead to high liver volumes, causing severe complaints. Livers can grow as large as 10 or 15 L, causing early satiety, decreased food intake and weight loss.^1^, ^5^, ^6^ Moreover, high intra‐abdominal pressure often leads to pain and umbilical and inguinal herniation.^6^, ^7^ Compression of the diaphragm can lead to dyspnoea.^7^ The large, protruding abdomen may also cause psychological problems because of a distorted body image and confronting inquiries about the possibility of pregnancy.^1^ These complaints often lead to a decreased quality of life in affected patients.^8^, ^9^ In the last 15 years, 1293 patients in Europe received a liver transplantation for PLD because of severe complaints and compromised physical functioning.^10^

### 1.1 An unmet need for new therapies

**FIGURE 1:**
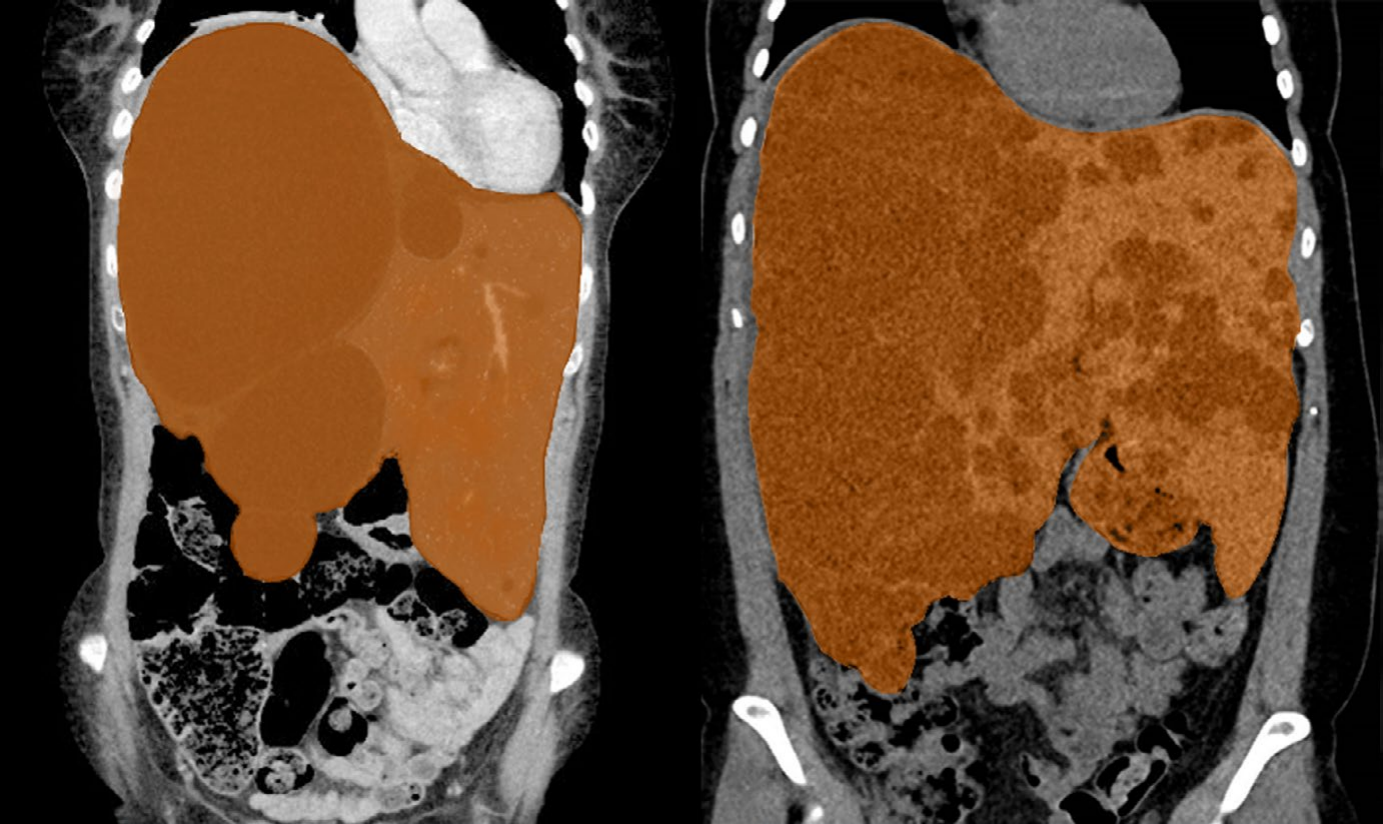
An example of localized polycystic liver disease with several large, dominant cysts (left panel) and diffuse polycystic liver disease with numerous smaller cysts (right panel), both resulting in hepatomegaly

Current treatment options in PLD can be subdivided into surgical and medical interventions.^1^, ^6^ Which option to choose depends on the size and distribution of liver cysts (Figure 1).^1^, ^6^ Surgical options, such as aspiration sclerotherapy, trans‐arterial embolization, laparoscopic cyst fenestration or hemihepatectomy, are suitable only for patients with several large dominant cysts, or cysts clustered in a few liver segments. Patients with massively enlarged liver volumes through numerous small cysts, distributed throughout the entire liver, are less suitable for these interventions.^1^, ^6^ If liver growth continues and in particular if liver‐related complications arise, these patients may need a liver transplantation.^11^ Treatment options to prevent liver growth and ultimately liver transplantation are limited.

The mainstay of treatment in patients with massive PLD is to halt natural growth using somatostatin analogues.^5^, ^12^ The clinical experience with somatostatin analogues in reducing cyst growth is mostly drawn from patients suffering from ADPKD and to a lesser extent from ADPLD patients.^5^, ^13^, ^14^ In ADPKD patients with PLD, 120 weeks of treatment led to a mean reduction in height‐adjusted total liver volume (hTLV) of 5.9%, but inter‐individual differences were large. In 39% of treated patients, liver growth continued.^5^ In addition, the effect of somatostatin analogues may decrease in the long term,^5^ there are questions on the safety (hepatic cyst infections, gallstones) of somatostatin analogues^3^, ^5^, ^15^ and it is unclear whether somatostatin analogues prevent liver transplantation in severely affected patients.^16^ Therefore, there is an unmet need for new therapies.

### 1.2 Estrogen, a new target for treatment?

In PLD, there is a large sex effect. Being female is the most important risk factor for the presence and severity of disease.^4^, ^11^, ^17^ Differences in disease severity between males and females could be caused by genetic differences, epigenetic differences and by differences in hormone levels. It is known that estrogen supplementation stimulates growth.^18^, ^19^ In addition, recent data suggest that liver growth does not follow a linear pattern with age, as was previously thought, but liver volumes spontaneously decrease after menopause in female patients.^20^ These data suggest that estrogens, or other female hormones, have an important effect on cyst growth. In this review, we summarize the epidemiological and preclinical evidence about the effect of female hormones on liver growth to answer clinical questions about estrogen supplementation, treatment decisions in postmenopausal patients and the potential of anti‐estrogenic treatment in PLD.

### 1.3 Methods

For this narrative review, literature was collected using a systematic approach. Literature was searched using Pubmed and EMBASE (keywords [estrogen AND polycystic liver], [cholangiocyte AND estrogen] and [cholangiocyte AND polycystic]). Sixty‐four articles were retrieved. All titles and relevant abstracts were read and 9 articles were selected for review.^18^, ^19^, ^21^, ^22^, ^23^, ^24^, ^25^, ^26^, ^27^ The other 53 were not relevant for this review, see Supplementary Table 1 for specific reasons. Of the 9 articles selected, 3 were experimental studies,^24^, ^26^, ^27^ 2 clinical epidemiological studies,^18^, ^19^ 3 review articles,^21^, ^23^, ^25^ and one a letter to the editor.^22^ Additional articles were obtained through citation snowballing to locate primary sources. This resulted in 9 experimental studies,^28^, ^29^, ^30^, ^31^, ^32^, ^33^, ^34^, ^35^, ^36^ 6 clinical epidemiological studies,^4^, ^11^, ^12^, ^17^, ^20^, ^37^ and 3 reviews. For a detailed description of the search strategy, see Supplementary Table 1.

## 2 FEMALE GENDER: THE MOST IMPORTANT RISK FACTOR FOR PLD

Female gender is the most important risk factor for the presence and severity of PLD.^17^ About 85% of PLD patients presenting at the outpatient clinic are females.^4^ On average, female patients are nine years younger at the time of diagnosis and 91% of female patients are symptomatic, compared to less than half of males (2,14). Liver volumes are larger in females than in men.^17^

Within women, the only other known risk factor for disease severity is disease type. On average, liver volumes in female patients affected by ADPKD are about 13% larger than in patients affected by ADPLD.^17^ In males, this association is absent.^17^

### 2.1 Which female hormones are responsible for the effect of gender in PLD?

It is difficult to distinguish which female hormones are responsible for the differences in PLD prevalence and severity between males and females. From several studies, it is clear that oestrogen exposure increases liver growth.^18^, ^19^ Estrogen‐containing contraceptives increase liver growth by 1.45% for every year used,^19^ and hormone replacement therapy in postmenopausal women leads to a liver growth rate of 7%/year compared to 2%/year in controls.^18^ Liver volumes tend to decrease after menopause^20^ and it has been proposed that liver growth accelerates during pregnancy.^37^ However, in most of these situations, there are changes in both estrogen and progesterone levels, and that hinders to pinpoint the individual contribution of progesterone on liver growth.

On an experimental level, many studies focused on the effect of estrogen on cystic proliferation.^24^, ^27^, ^28^, ^29^, ^30^ Only four studies targeted other female hormones.^32^, ^33^, ^34^, ^36^ Based on the currently available research, the role of estrogens has become clear but an additional effect of other female hormones cannot be ruled out.

### 2.2 What is the effect of estrogen on cystic proliferation in animal or experimental studies?

Human hepatic cysts are lined by cholangiocytes and most experimental studies have been performed using bile duct‐ligated rats. In these rats, ligation of the bile duct induces a selective proliferation of cholangiocytes, resulting in an increase in the size of intrahepatic bile ducts.^28^

Estrogen receptors alfa and beta (ER‐α and ER‐β) are present on cholangiocytes in normal rats and up‐regulated in bile duct‐ligated rats.^29^ ER‐α was present on 9% of cholangiocytes in normal rats and 25% of cholangiocytes of bile duct‐ligated rats (*P* = .03). ER‐β was present on 3% of cholangiocytes in normal rats and 80% of cholangiocytes in bile duct‐ligated rats (*P* < .0001).^24^, ^29^, ^30^

Treatment with anti‐estrogen therapy, either tamoxifen or fulvestrant, in 3‐week‐old bile duct‐ligated rats decreased the biliary mass 2.5 fold (*P* < .01). However, their bile duct mass was still higher compared to normal rats.^29^ Ovariectomy in female bile duct‐ligated rats led to a 30% lower bile duct volume than in those without ovariectomy (*P* = .02).^30^ Treatment with fulvestrant of rats after ovariectomy decreased bile duct mass even further. Addition of 17β‐estradiol in this population increased bile duct mass to similar volumes as seen in the bile duct‐ligated rats without ovariectomy.

**FIGURE 2:**
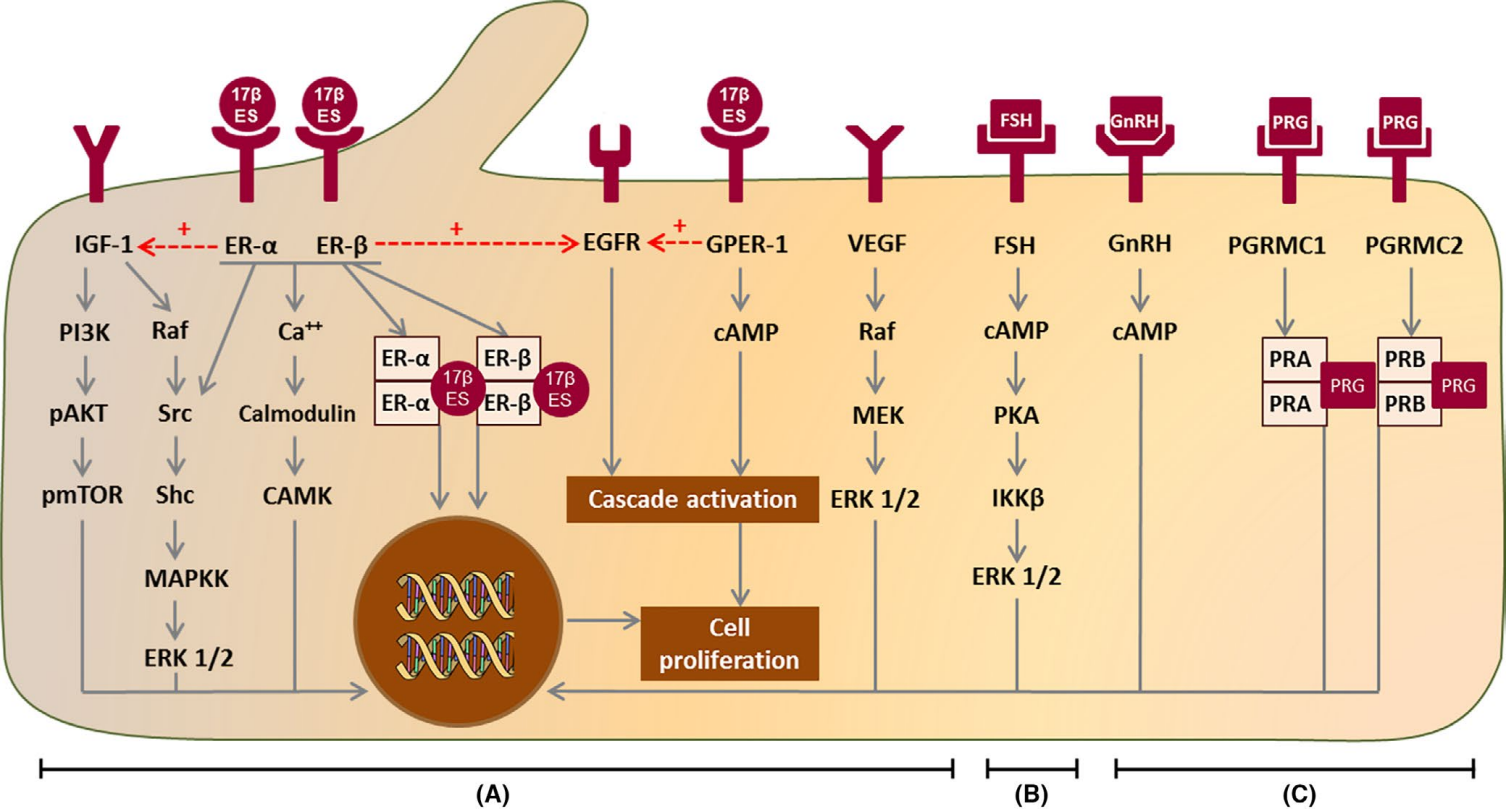
Summary of estrogen, progesterone, FSH and GnRH associated growth pathways in polycystic liver disease. A, Pathways that are found in bile duct‐ligated rats and confirmed in human hepatic cyst cells. B, Receptors and associated pathways studied in human hepatic cyst cells only. C, Receptors and pathways studied in bile duct‐ligated rats only. The growth receptors IGF‐1, VEGF and EGFR are present on the cell membrane and induce proliferation. IGF‐1 receptor induces proliferation directly, but is also indirectly involved in estrogen‐induced proliferation. The ER‐α and ER‐β are thought to be a nuclear receptors that have genomic effects, but are also found as epithelial receptor in ADPKD hepatic cysts cells, and induce proliferation via non‐genomic pathways such as the Src‐Shc‐MAPKK‐ERK1/2 pathway and via Ca^++^. Next to the ER‐α and ER‐β receptor, a third estrogen receptor, the G‐coupled Protein Estrogen Receptor 1 (GPER‐1) is present on the cell membrane (work in progress from our own group). The EGFR is possibly activated by activation of the ER‐α and ER‐β as well as by the GPER‐1. The FSH receptor stimulates cell proliferation via cAMP related pathways. In bile duct‐ligated rats, GnRH receptors are found en GnRH induces proliferation. However, it is unclear whether this is a direct effect or most effects are related to enhanced production of other female hormones. Progesterone membrane receptors are present on the cell membrane (PGRMC1 and PGRMC2) and the nucleus (PRA and PRB)

The stimulation of cholangiocyte growth by estrogens is thought to be partly caused by the p‐ERK1/2 pathway and the protein Shc.^31^ Inhibition of cholangiocyte proliferation by tamoxifen or fulvestrant reduced ERK‐1/2 and Shc. Adding estrogens led to an increased expression of both pathways.^31^ Furthermore, there is an interplay between estrogen and insulin‐like growth factor (IGF). Probably, ERs in the plasma membrane play an important role in this, since it is known that they can associate with signalling proteins such as Shc and Src directly, and they can interact with and activate the IGF‐1 receptor (Figure 2).^38^ Next, estrogens seem to promote secretion^30^ and could stimulate cholangiocyte proliferation by their effect on the immune system, especially via macrophages.^39^, ^40^ It is also possible that estrogens have an effect on angiogenesis by interaction with VEGF.^41^

**FIGURE 3:**
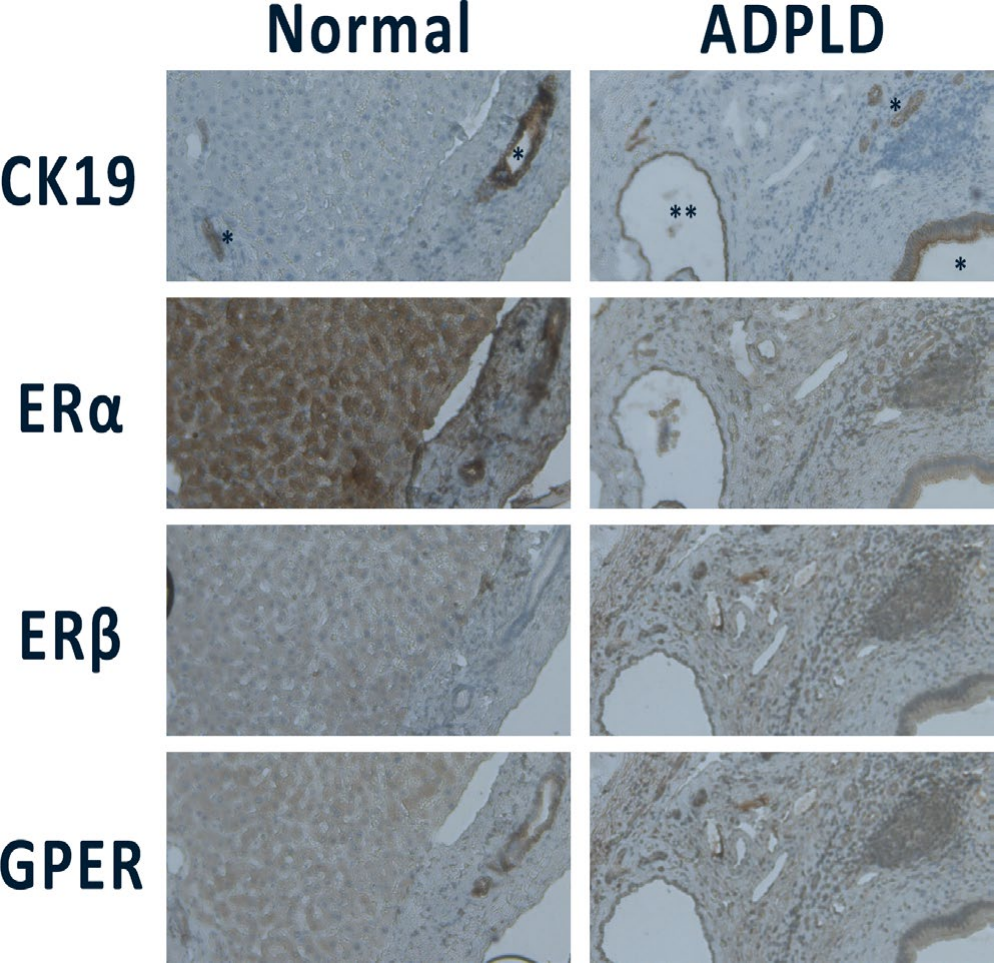
Immunohistochemistry images for CK19, ER‐α, ER‐β and GPER in normal liver and ADPLD hepatic cysts from separate patients. CK19 (cytokeratin 19) is a marker for cholangiocytes. In this tissue, we recognize lesions with cuboidal cholangiocytes such as Von Meyenburg complexes (*) and one cyst with flat epithelium (**). In this ADPLD‐patient, all cholangiocytes stained positive for ER‐α, ER‐β and GPER

In humans, the estrogen receptors ER‐α and ER‐β are present on the surface of polycystic liver cells in ADPKD as well as ADPLD (Figure 3).^23^, ^27^ Immunohistochemistry on liver tissue from 6 patients with ADPKD and 4 controls showed that normal intrahepatic bile ducts are negative for ER‐α and ER‐β in both patients and controls. Hepatic cyst epithelium and reactive bile ducts close to the cysts are positive for both estrogen receptors ER‐α and ER‐β.^27^ Immunohistochemistry for insulin‐like growth factor 1 (IGF1) had a similar distribution. This supports the earlier proposed pathway, in which estrogens and IGF1 together promote cyst growth.^23^, ^24^, ^27^

The effect of addition of estrogen and estrogen receptor blockers was studied in an immortalized cell line, derived from hepatic cyst cells in ADPKD patients (LCDE cell line). After depletion of serum for 24 hours, administration of estradiol leads to an increase in cholangiocyte proliferation. Administration of IGF1 also leads to increased proliferation. If a selective IGF1 blocker was used, IGF1‐induced proliferation was completely blocked. If an estrogen blocker, such as fulvestrant was used, estrogen‐induced proliferation was partially blocked, but if an estrogen blocker and IGF1 blocker were used after administration of estrogen, proliferation was completely blocked. This indicates that estrogen indeed induced proliferation via IFG1.^23^, ^27^

In addition to the presence of the ER‐α and ER‐β, the G‐coupled protein oestrogen receptor 1 has also been discovered recently.^42^ Work from our own group shows that the GPER1 receptor is present on hepatic cyst cells in human ADPLD tissue (Figure 3).

## 3 EFFECT OF ESTROGEN IN CLINICAL PRACTICE

Animal and experimental studies show the effect of curtailing or stimulating estrogen signalling in bile duct‐ligated rats and human cell lines. To better understand the clinical importance of estrogen in PLD, we can learn from clinical situations where patients are exposed to estrogen (contraceptives, hormone replacement therapy) or estrogen depletion (postmenopausal status).

### 3.1 What is the effect of estrogen supplementation in PLD patients?

Gabow et al proposed already in 1990 that liver growth and estrogen exposure were possibly related.^37^ Later, in a case‐control study including 19 ADPKD patients with polycystic livers, liver growth was compared between postmenopausal patients with and without hormonal supplementation therapy. One year of treatment with hormone replacement therapy led to a mean liver growth rate of 7%/year, while liver volumes declined in the group without treatment, with a mean rate of −2%/year.^18^

These data are contradicted by recent large retrospective studies. These studies do not confirm this correlation between liver size and estrogen‐containing contraceptives.^20^, ^43^ Possibly, this contradiction can be explained by the fact that these studies included mostly mild PLD patients and the dosage of estrogen in oral contraceptives decreased considerably in the last decades, from 100 mcg to less than 30 mcg.^1^ On the other hand, a cross‐sectional study suggested that even the currently low‐dose oestrogen‐containing oral contraceptives provoke liver growth. Every year of exposure was associated with a 1.45% larger liver volume in pre‐menopausal patients.^19^

### 3.2 Does pregnancy affect PLD?

Some studies suggest a correlation between the history of pregnancy and the number of pregnancies and disease severity.^37^ However, these findings are not consistent among studies.^19^, ^20^, ^43^ Clinical observations suggest that there is increased growth during pregnancy. It is unknown whether liver growth rates accelerate during pregnancy, or complaints increase due to the higher intra‐abdominal volume. These inconsistent results could be explained by the observed increase in estrogens during pregnancy, but prolonged reduction in endogenous estrogens thereafter, resulting in a similar or even lower life‐time estrogen exposure in parous women compared to nulliparous women. ^19^

### 3.3 What is the effect of estrogen depletion on PLD patients?

As far as we know, the effect of medications that block the production of estrogen or oestrogen receptors has never been studied in PLD patients. The only physiological situation that comes with low estrogen and progesterone levels is the postmenopausal status. Many epidemiological studies have demonstrated that liver volumes as well as liver growth rates decrease after menopause (Table 1).

**TABLE 1:** Liver volumes and liver growth rates in female patients before and after menopausal age in several cross‐sectional, observational and interventional studies

Author, year	Study aim		Population	Age cutoff (year)	Subgroup	Age cut‐off		*P* value
						Mean (change in) TLV/hTVL younger	Mean (change in) TLV/hTVL older	
Cross‐sectional studies								
Van Aerts (2019)		Effect of oral contraceptives on liver growth	n = 287 ADPKD 63% ADPLD 37%	51	Females, n = 287	TLV 3968 ml	TLV 3047 ml	.001
Van Aerts (2018)		Association between disease (ADPKD vs ADPKD) and severity	n = 360 ADPKD 67% ADPLD 33%	51	Females, n = 291	hTLV 2485 ml	hTLV 1925 ml	.001
					ADPKD, n = 191	*Not given*	*Not given*	.003
					ADPLD, n = 100	*Not given*	*Not given*	.41
Longitudinal studies								
Chebib et al (2016)		Association between PKD mutation and liver volume	n = 211 ADPKD 100%	48	Females, n = 128	2.65%/yr	0.09%/yr	.037
					Females with severe PLD, n = 28	75% progressors: 10.6%/yr^a^ 25% non progressors: −2.4%/yr^a^	42% progressors: 4.9%/yr^a^ 58% non progressors: −2.4%/yr^a^	*Not given*
Gevers et al (2013)		Meta‐analysis of three small somatostatin analogue trials	n = 107 ADPKD 72% ADPLD 28%	48	Females in placebo arms, n = 35	4.8%	0.6%	*Not given*
Aerts (2019)		Lanreotide for PLD	N = 157 ADPKD 100%	45	Males and females, n = 157	6.42%	2.58%	*Not given*

Abbreviations: ADPKD, autosomal dominant polycystic kidney disease; ADPLD, autosomal dominant polycystic liver disease; hTLV, height‐adjusted total liver volume; TLV, total liver volume.

^a^ These values are no mean but median.

**FIGURE 4:**
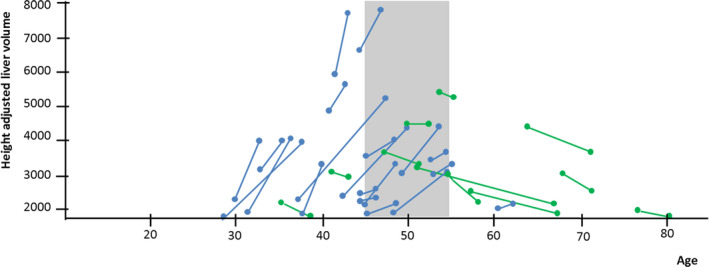
Liver volumes increase before, and decrease after menopause in female patients with large liver volumes suffering from polycystic liver disease. Change in height‐adjusted liver volume in the 32 female ADPKD patients with baseline height‐adjusted liver volume >1700 ml/m, showing that liver volume in most cases increases before, and decreases after menopause. Age of menopause in 95% of polycystic liver disease patients is between 45 and 55 years (the grey shaded area). This figure is adapted by the authors of this review from the figures in *Chebib et al (2016)* to show more clearly what the effect of menopause may be

The most striking example of decreased liver growth after menopause is the recent retrospective cohort study that collected data on liver growth rates in 211 patients with ADPKD and PLD^20^ (Figure 4). In female patients <48 years, the median liver growth was 2.65%/year, while this was 0.09%/year in female patients >48 years (*P* = .037). In female patients with large polycystic livers (defined as hTLV >1800 ml, n = 28), differences were even larger. In these patients, 75% of patients <48 years showed growth of the liver (plus 10.6%/year), compared to 32% of patients >48 years (plus 4.9%/year). In 58% of patients >48 years, liver volumes decreased 2.4%/year. In male patients, age did not have a modifying effect on liver growth rates before and after the age of 48 years, corroborating the concept that this change in female patients may be caused by changes in female sex hormones.^20^ Finally, data from several intervention trials with somatostatin analogues are consistent with the finding that liver growth rates are higher in younger women, and decrease after menopause.^12^, ^13^, ^44^, ^45^ An overview of these data is shown in Table 1.

### 3.4 Is the effect of estrogen on liver growth different for ADPKD and ADPLD?

The effect of female hormones on PLD may differ between ADPKD and ADPLD, most likely in view of differences in genetic defects.^46^ Although ADPKD and ADPLD are caused by different mutations, mutations in both diseases affect the presence of functional polycystin 1 and 2 and suggesting a shared pathophysiology.^47^ ER‐α and ER‐β are present on ADPKD as well as ADPLD cyst cell lines (Figure 3).^23^, ^27^

In one study, ER receptors were absent from ADPLD cell lines, but corroborating data were not shown.^48^ Preliminary data from our own group suggest that ER‐α and ER‐β are present also on ADPLD‐derived cells (Figure 3). Up‐regulation of oestrogen receptors is seen in several biliary diseases, such as primary biliary cholangitis and cholangiocarcinoma.^24^ Enhanced ER expression might be a response to injury rather than a causative mechanism. Only the G‐coupled protein estrogen receptor (GPER) was present on APDLD cells (Figure 3), while its presence on APDKD cells is unknown.

When it comes to epidemiological studies, most data are derived from ADPKD patients.^18^, ^20^, ^37^, ^43^ In the study by van Aerts et al, liver volumes of both 191 ADPKD and 100 ADPLD patients looked lower in patients older than 51 years of age, but this only reached statistical significance in the ADPKD group.^17^ Data from Chebib et al, that show a decrease in liver volumes after menopause, are obtained in ADPKD patients. In trials on somatostatin analogues, the majority of patients suffered from ADPKD, making it difficult to find data on growth rates restricted to ADPLD patients.^5^, ^12^ However, the association between oestrogen exposure and disease severity has also been demonstrated for ADPLD.^4^, ^11^, ^19^ Altogether, we hypothesize that estrogens also play a role in ADPLD.

### 3.5 Are there other female hormones that could affect polycystic liver growth?

As mentioned previously, most data on female hormones in PLD are on oestrogens. Epidemiological data or in vivo studies for other female hormones such as progesterone, Follicle Stimulating Hormone (FSH) or Gonadotropin Releasing Hormone (GnRH) are absent. On experimental level, only a couple of studies have been performed.

One study assessed the effect of progesterone in bile duct‐ligated rats. Progesterone receptors PGRMC1 and PGRMC2 were present on cholangiocyte membranes and cholangiocyte proliferation increased after administration of progesterone. Administration of anti‐progesterone decreased intrahepatic biliary mass. Although there is no research available that confirms these findings in PLD patients, a potential effect of progesterone on liver growth cannot be ruled out.^32^

Follicle Stimulating Hormone Receptors are found on human hepatic cyst cells, with a correlation between the degree of expression and cyst size. Proliferation increased after addition of Follicle Stimulating Hormone. After addition of a FSH blocker, proliferation was partially blocked. Also in bile duct‐ligated rats, FSH stimulation induced enhanced cholangiocyte proliferation and increased cAMP levels and ERK phosphorylation. Again, it is unclear whether FSH exerts an effect on liver growth in vivo.^33^, ^34^

Last, GnRH receptors (GnRHR) are found on cholangiocytes of bile duct‐ligated rats. Administration of GnRH to bile duct‐ligated rats enhanced bile duct mass. It has been proposed that GnRH induces proliferation directly but it cannot be excluded that increased proliferation is caused by enhanced production of other female hormones via GnRH.^36^

Altogether, most evidence is available for an effect of oestrogens in PLD, but an additional effect of other female hormones cannot be ruled out.

## 4 IMPLICATIONS FOR CLINICAL PRACTICE

### 4.1 What should we advise our patients about estrogens?

In general, there is a wide consensus to discourage female PLD patients to take estrogen‐containing contraceptives or use hormone replacement therapy.^1^, ^4^, ^6^, ^18^, ^19^, ^22^, ^47^, ^49^, ^50^ Little is known on the effect of oral contraceptives containing only progesterone. We cannot rule out that progesterone stimulates liver growth, either directly or through synergism of estrogen. The epidemiological studies offer little help in this respect and it is not possible to distinguish the contribution of individual components of oral contraceptives. The uncertainty about the effect of progesterone on disease progression should be weighed against the need for contraception in clinical care. Little is known about hormone containing intrauterine devices, but the hormone dosages are lower than oral forms.^51^ Of course, barrier contraception methods, surgical sterilization or a copper intrauterine device do not contain female hormones and are a safe choice.

### 4.2 How should we treat postmenopausal patients?

We described several studies that show that liver growth rates are lower and (in some cases) liver volumes decrease after menopause in female PLD patients.^18^, ^52^ In cases where somatostatin analogues are prescribed in postmenopausal patients, it seems useful to evaluate liver growth to monitor whether the (expensive) somatostatin analogues should be continued, in view of a possible deviation from the natural course of disease after menopause. If female patients are listed for liver transplantation with liver volume‐related complaints as the primary indication, it is also relevant to follow liver volumes regularly to assess liver growth. According to the current data, the natural course in liver volume could be on a downward trajectory at the time of liver transplantation since the mean age of liver transplantation is 52 years, when most patients are postmenopausal.^11^, ^19^

### 4.3 How can estrogen be a target for future therapies?

The summarized experimental and epidemiological findings suggest that female hormones could be a promising target for new therapy.^7^, ^24^, ^47^ Such a treatment would only be suitable for female patients, but >80% of patients with severe PLD are females.^4^

**FIGURE 5:**
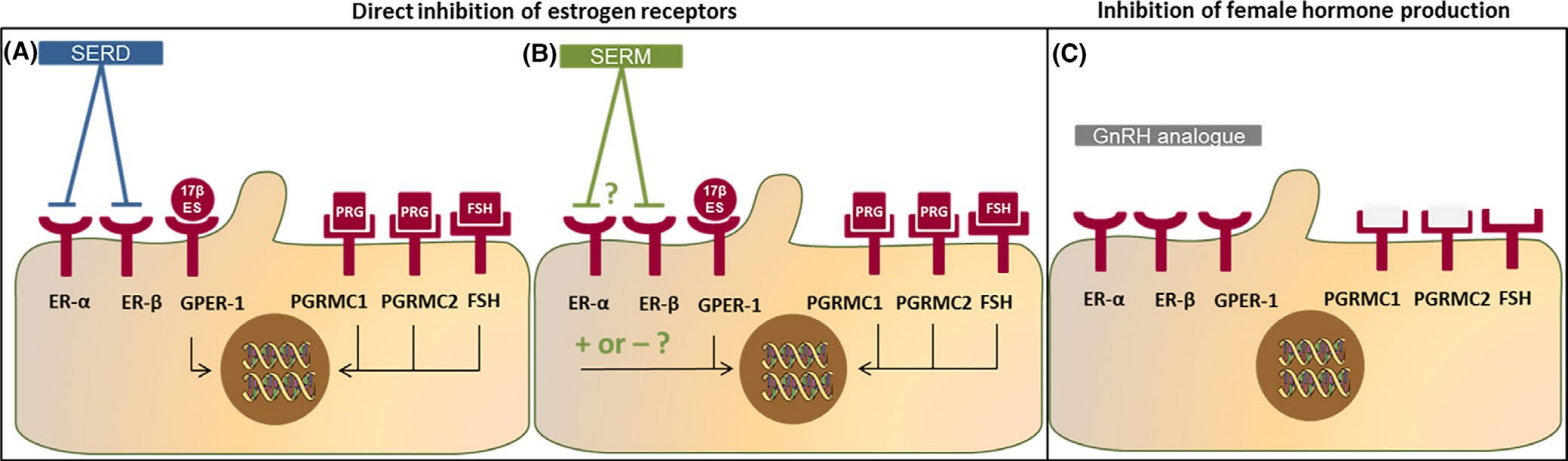
Schematic overview of different approaches to inhibit estrogen and other female hormones in polycystic liver disease. A, Using a SERD (selective estrogen receptor degrader) the ER‐α and ER‐β are blocked. However, oestrogen could still have effect via the GPER‐1 receptor and other female hormones could also influence cholangiocyte proliferation. B, It is unclear whether a SERM (selective estrogen receptor modulator) would have antagonistic of agonistic effects on ER‐α and ER‐β on human cholangiocytes. A SERM would have no effect on the GPER‐1 receptor or receptors for other female hormones. C, A GnRH analogue blocks the production of the female hormones estrogen, progesterone, LH and FHS. Therefore, the receptors for female hormones will not be activated

Targeting female hormones, and especially the estrogen pathway, could be approached in two ways (Figure 5). The first and most direct approach is to block the estrogen receptors alfa and beta. This could be done using selective estrogen receptor modulators (SERM), of which tamoxifen is the most well known. However, these SERM can have either agonistic or antagonistic effects on estrogen receptors, depending on the tissue. In case of breast cancer, SERMs act as an antagonist, but in other tissues, endometrium for example, SERMs act as an agonist, increasing the risk of endometrium cancer. So far, it is unclear whether SERMs act as an agonist or antagonist on human cholangiocytes.

Another option to block the estrogen receptors are selective estrogen receptor degraders (SERD), with fulvestrant as an example. This class of drugs degrades the estrogen receptor, acting as an antagonist independent of tissue type, but can cause venous thrombo‐embolism as a side effect and are not suitable for patients with a eGFR<30 ml/1.73m/min, which may be an issue in ADPKD patients.^53^ It is also uncertain whether SERDs could block the GPER‐1 receptor, which we showed to be present, at least on ADPLD cyst tissue (Figure 3).

**FIGURE 6:**
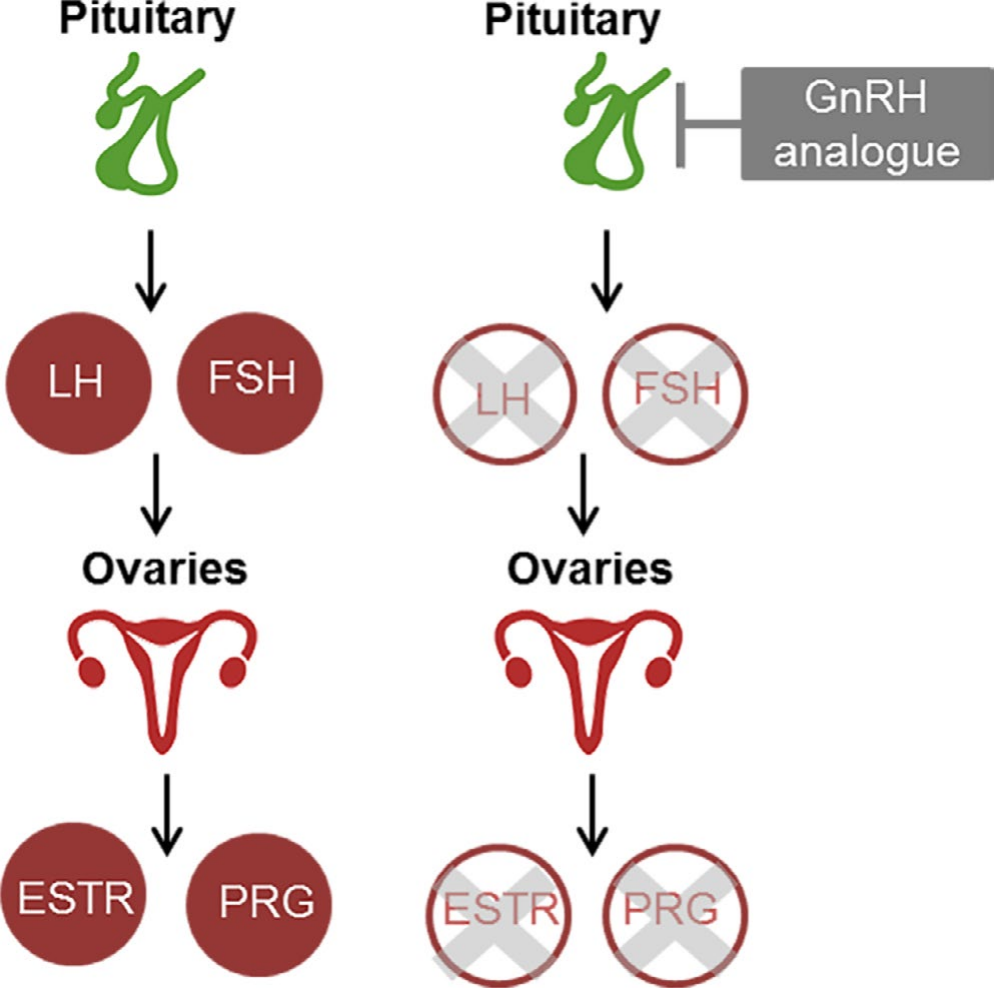
Schematic overview of the effect of GnRH analogues on the production of female hormones. On the left, the normal situation in premenopausal women is shown. The hypothalamus produces GnRH, which stimulates the pituitary to produce LH and FSH. These hormones stimulate the ovaries to produce estrogen and progesterone. A GnRH analogue stimulates the pituitary so heavily, that desensitization occurs, a no more LH and FSH is produced. Therefore, the ovaries will not produce estrogen and progesterone anymore

The second approach could be to target the production of female hormones systemically, using Gonadotropin Releasing Hormone (GnRH) antagonists or GnRH analogues. Antagonists block the production of GnRH, and thereby the whole cascade producing FSH, LH, oestrogen and progesterone directly. However, they are expensive and only available using daily injections, making it less suitable for clinical practice. GnRH analogues stimulate the hypothalamus to produce GnRH in such a strong way that it will lead to desensitization in a couple of weeks, yielding an increase in GnRH on the short term, but having the same effect as GnRH antagonists on the long term (Figure 6). GnRH analogues are relatively cheap and available in 3 monthly depot injections. In this approach, not only estrogen but also progesterone and FSH are lowered, which probably also stimulate liver growth. Disadvantages are side effects related to the hypo‐estrogenic status, such as hot flashes, and if used chronically on the long term, a slightly higher risk of cardiac events and a higher risk of osteoporosis.^54^, ^55^

## 5 CONCLUSION

Experimental as well as epidemiological evidence suggests an important role for female hormones, especially oestrogen, in PLD. estrogen receptors alfa and beta are present on the surface of human hepatic cyst‐derived cells. These cells proliferate after addition of estradiol, and proliferation decreases after addition of an estrogen receptor blocker. The majority of PLD patients is female, female patients are affected more severely and oestrogen‐containing contraceptives or hormone replacement therapy lead to more severe disease progression. Clinical studies show that on average, hepatic growth rates are lower in females after menopause, and in some patients, liver volumes decrease after menopause.

Especially in patients suffering from massive and progressive PLD, there is a large unmet need for new treatments. Given these data, inhibition of female hormones could be a promising target for new therapies. The mechanisms of action, activation and blockage of estrogen receptors are complex and the effect of selective estrogen receptor modulators is difficult to predict. Three different estrogen receptors are involved (ER‐α, ER‐β and GPER‐1) and other female hormones may also play a role. Therefore, we argue that proof‐of‐concept studies targeting female hormones should start with a GnRH analogue. It seems time to start a trial using this promising intervention.

## TRIAL REGISTER NUMBER

Not applicable.

## CONFLICT OF INTERESTS

The authors received unrestricted grants from the Dutch Government (ZonMW grant 10140261910001) and Abbvie. The organization had any role in neither the conception nor in writing of this manuscript. Dr Gansevoort received grant support and fees for serving on advisory boards and steering committees from Galapagos, IPSEN, Otsuka Pharmaceuticals and Sanofi‐Genzyme. In addition, Dr Gansevoort holds the Orphan Medicinal Product Designation status at the European Medicines Agency for lanreotide as treatment for kidney function decline in ADPKD (EMA/OD/027/15). Dr Drenth has received grant support and fees for serving on advisory boards and consultancy from IPSEN and Novartis. All money is paid to their institutions. No other potential conflict of interest relevant to this article was reported.

## Supporting information

Table S1
