# Supplementary figures and images for: Estrogens in polycystic liver disease: A target for future therapies?
Source: Liver Int. 2021 Jul 10;41(9):2009–19. doi: 10.1111/liv.14986 (PMC8456902; doi:10.1111/liv.14986)

## SUPPLEMENTARY MATERIAL

Supplementary table 1. Selection of articles for review.

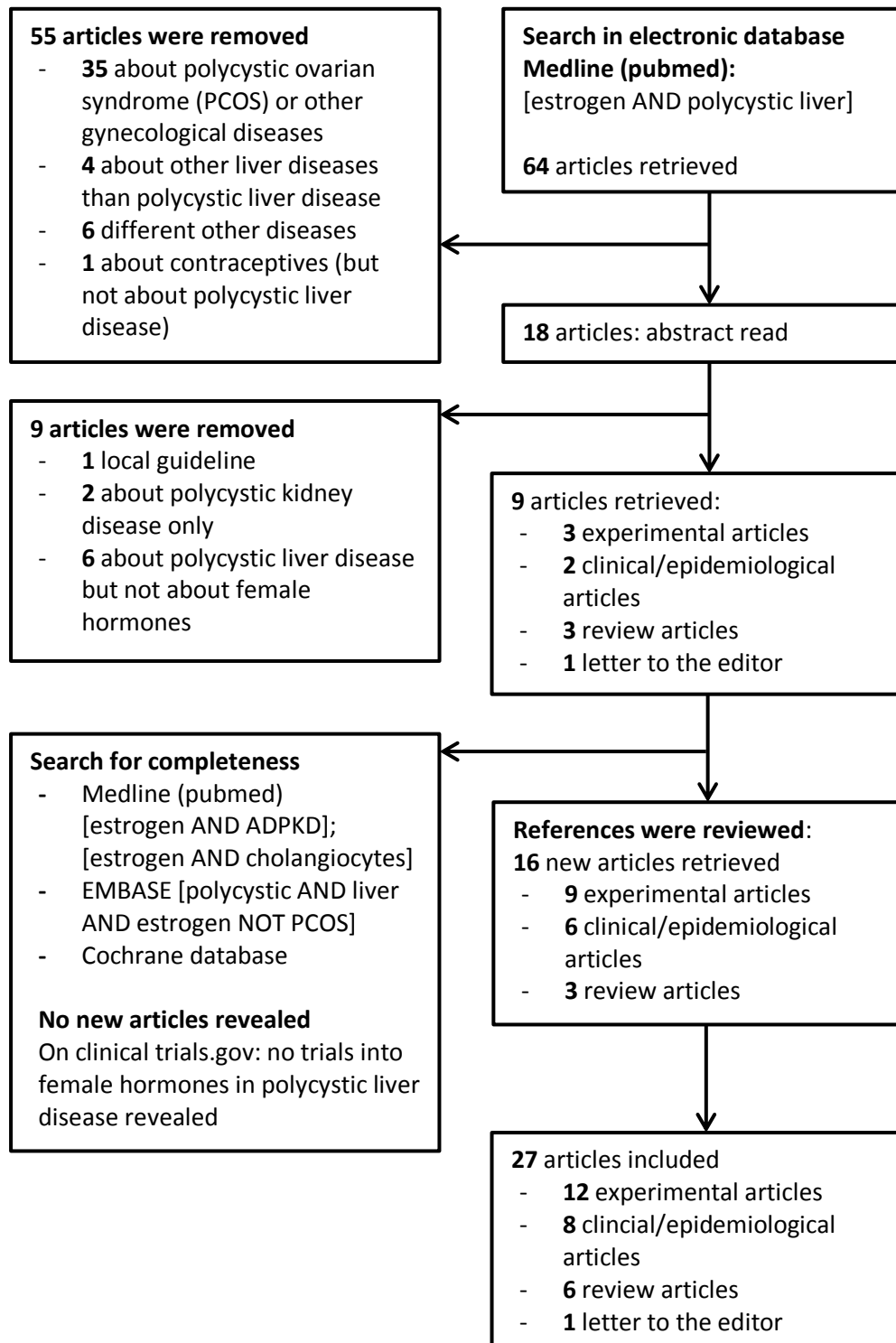

Supplement: Supplementary file 1 — Table S1 [file LIV-41-2009-s001.pdf]
